# The *WAKL10* Gene Promotes Flg22-Triggered Immunity by Interacting with FLS2 and BAK1 in *Arabidopsis*

**DOI:** 10.3390/genes17050561

**Published:** 2026-05-09

**Authors:** Lu Zhang, Jiale Gao, Lingya Yao, Yunxia He

**Affiliations:** 1 School of Environmental and Chemical Engineering, Shanghai University, Shanghai 200444, China; 2 Shanghai Key Laboratory of Plant Molecular Sciences, College of Life Sciences, Shanghai Normal University, Shanghai 200234, China

**Keywords:** flg22-triggered immunity, wall-associated kinase, FLS2-BAK1 receptor complex, plant disease resistance

## Abstract

**Background/Objectives:** The wall-associated kinases (WAKs) and WAK-like proteins (WAKLs) comprise a unique receptor-like kinase subfamily in plants, which have been shown to regulate plant development and defense responses by sensing cell wall-derived components, such as pectin or pectin fragments. In this study, we aimed to characterize the function of *WAKL10* in flg22-triggered immunity in *Arabidopsis*. **Methods:** Through functional analyses of *WAKL* genes in *Arabidopsis*, we identified *WAKL10* as the most pronouncedly induced *WAKL* member in response to flg22 treatment. Gain- and loss-of-function genetic analyses were performed to assess its role in flg22-triggered immune responses, including mitogen-activated protein kinase (MAPK) activation, reactive oxygen species (ROS) burst, and defense gene induction. Transgenic *Arabidopsis* plants expressing a kinase domain-deleted mutant (*WAKL10-ΔK*) were generated. Co-immunoprecipitation assays were conducted to examine interactions with FLAGELLIN-SENSITIVE 2 (FLS2) and BRI1-ASSOCIATED RECEPTOR KINASE 1 (BAK1). Heterologous overexpression of *WAKL10* in tomato was also tested for bacterial disease resistance. **Results:** *WAKL10* positively regulates flg22-triggered immune responses. Interestingly, *WAKL10-ΔK* retains the capacity to potentiate these responses. Co-immunoprecipitation assays showed that both wild-type WAKL10 and WAKL10-ΔK constitutively associate with FLS2 and BAK1. Overexpression of *WAKL10* in tomato confers enhanced bacterial disease resistance. **Conclusions:** The extracellular domain of WAKL10 promotes FLS2-BAK1 complex formation, thereby contributing to flg22 signaling. This study reveals a new function of WAKLs, distinguished from their proposed role in sensing cell wall components. The functional conservation of WAKL10 suggests its potential application in engineering disease resistance in crop plants.

## 1. Introduction

Plants have a sophisticated and multi-layered immune system that enables their survival in pathogen-rich environments while maintaining growth and development [1]. This plant immune system is built upon two interconnected defense tiers: pattern-triggered immunity (PTI) and effector-triggered immunity (ETI) [2,3]. PTI acts as the first line of plant immunity and is launched when plasma membrane-resident pattern recognition receptors (PRRs) perceive conserved microbial signatures, namely pathogen-associated molecular patterns (PAMPs) from microbes or endogenous damage-associated molecular patterns (DAMPs), that are liberated from host tissues following wounding [4,5,6]. In contrast, ETI involves intracellular nucleotide-binding leucine-rich repeat (NLR) receptors that recognize pathogen effector proteins, typically triggering stronger responses including hypersensitive cell death [7,8,9,10].

In plants, pattern recognition receptors (PRRs) fall into two main classes: receptor-like kinases (RLKs) and receptor-like proteins (RLPs) [11,12]. RLKs feature extracellular domains that perceive ligands and intracellular kinase domains responsible for transducing signals [13,14]. In contrast, RLPs lack such intracellular kinase domains and therefore must associate with co-receptors to initiate signaling [15,16]. In *Arabidopsis thaliana*, well-studied PRRs include the leucine-rich repeat receptor-like kinases (LRR-RLKs) FLS2 and EFR (EF-Tu receptor), which specifically recognize the flagellin-derived peptide flg22 and the elongation factor Tu epitope elf18, respectively [17,18,19].

The extracellular domains of PRRs display remarkable structural diversity, including LRR domains for protein ligand binding, lysin motif (LysM) domains for chitin oligosaccharide recognition [20], lectin domains for carbohydrate perception [21], and wall-associated domains for sensing cell wall components [22]. This structural versatility enables plants to establish comprehensive surveillance networks against diverse microbial threats. Upon PAMP binding, PRRs typically associate with co-receptors such as BAK1/SERK (Somatic embryogenesis receptor-like kinase) family members, forming active complexes to activate downstream signaling cascades [23,24]. These cascades elicit cellular responses, including calcium influx, reactive oxygen species (ROS) burst, mitogen-activated protein kinase (MAPK) activation, and coordinated activation of defense hormone pathways, ultimately reprogramming defense gene expression to establish effective barriers against pathogen invasion [25,26].

Among various PRR families, the wall-associated kinases (WAKs) and the related WAK-like kinase (WAKLs) comprise a unique receptor-like kinases subfamily functioning as critical sensors of cell wall integrity and mediating cell wall-cytoplasmic communication [27,28]. The WAK/WAKL family identified in *Arabidopsis* consists of 5 WAKs and 22 WAKLs. Among *Arabidopsis* WAK family members, WAK1 has been shown to recognize the DAMP molecule oligogalacturonide derived from cell wall pectin to trigger PTI responses and mediate resistance against the necrotrophic fungal pathogen *Botrytis cinerea* [29]. In this study, to comprehensively investigate the functions of *WAKLs* in *Arabidopsis* immunity, we characterized the expression patterns of *WAKL* genes in response to flg22 treatment and identified *WAKL10* as the most strongly induced *WAKL* member by flg22. Further genetic analyses using *WAKL10*-overexpressing plants and knockout mutants indicated that WAKL10 positively regulates flg22-induced immune responses, while co-immunoprecipitation assays showed that WAKL10 constitutively associates with both the flg22 receptor FLS2 and the co-receptor BAK1. Subsequent functional domain analysis indicated that the intracellular kinase domain of WAKL10 is dispensable not only for its function in potentiating flg22-triggered immune responses but also for its association with FLS2 and BAK1. These data suggest that WAKL10 relies on its extracellular domain to promote the assembly of the FLS2-BAK1 complex for flg22 perception, which represents a new function of WAKLs and is distinguished from the proposed role of WAKLs in sensing cell wall components. Moreover, overexpression of *WAKL10* significantly increased bacterial resistance in transgenic tomato, indicating the promising application of *WAKL10* in the engineering of crops for improved disease resistance.

## 2. Materials and Methods

### 2.1. Plant Materials and Growth Conditions

Wild-type plants used in this study consisted of *Arabidopsis* ecotype Columbia-0 (Col-0) and tomato (*Solanum lycopersicum*) cultivar Micro-Tom. The T-DNA insertion line *wakl10-1* (SALK_132887) was acquired from the *Arabidopsis* Biological Resource Center (ABRC).

To analyze gene expression, assess MAPK activation, and measure ethylene production, we cultivated *Arabidopsis* and tomato seedlings on half-strength (1/2) Murashige and Skoog (MS) plates at 22 °C for 7 days. Growth took place in an incubator under continuous light (60 μE m^−2^ s^−1^). Subsequently, the seedlings were transferred to liquid 1/2 MS medium inside gas chromatography (GC) vials and incubated for an additional 7 days under the same light conditions. After this period, the seedlings in GC vials were exposed to either 10 µM estradiol (Est), 100 nM flg22, 300 nM flgII-28, or their corresponding solvent controls (0.1% DMSO for Est; water for flg22 and flgII-28). At designated time points following treatment, ethylene production was quantified, and seedlings were harvested for Quantitative real-time PCR (RT-qPCR) or immunoblotting. For assays requiring soil-grown plants, we used 4-week-old *Arabidopsis* and tomato plants. These were raised at 22 °C in a growth chamber under a 14-h light/10-h dark photoperiod and served for protoplast isolation, reactive oxygen species (ROS) measurement, and pathogen resistance tests.

### 2.2. Generation of Recombinant Constructs and Transgenic Plants

The *WAKL10* coding sequence (CDS) was PCR-amplified from the total cDNA of Col-0 plants. For stable plant transformation, the *WAKL10* CDS was cloned into the vector *pER8-Est:HA* to generate the estradiol-inducible *Est:WAKL10-HA* expression construct. For transient expression in protoplasts, the *WAKL10* CDS was cloned into the vector *pHBT-35S:FLAG* to generate the *35S:WAKL10-FLAG* construct. The DNA fragment encoding the kinase domain-deleted mutant *WAKL10-ΔK* was PCR-amplified from *WAKL10* CDS and subsequently cloned into both the *pER8-Est:HA* and *pHBT-35S:FLAG* vectors to generate the *Est:WAKL10-ΔK-HA* and *35S:WAKL10-ΔK-FLAG* constructs for plant and protoplast transformation, respectively. For co-IP assays, the *FLS2* and *BAK1* coding sequences were PCR-amplified from the Col-0 total cDNA and then cloned into the vector *pHBT-35S:HA* to generate *35S:FLS2-HA* and *35S:BAK1-HA* constructs. All the primer sequences used for the generation of recombinant constructs are listed in Supplementary Table S2.

Transgenic Arabidopsis lines were produced in the Col-0 background via *Agrobacterium*-mediated floral-dip transformation [30]. For each construct, approximately 50 T1 plants were assayed for transgene expression by immunoblotting using an anti-HA antibody. For each construct, two independent T2 lines carrying a single T-DNA insertion (determined via segregation analysis on hygromycin-containing plates) and showing relatively high transgene expression were selected for subsequent experiments. Owing to space constraints in the main figures, data from one representative line are presented, as they typify the observed phenotypes across independent transformants.

### 2.3. Bioinformatic Analysis of the WAKL Family RLKs

Analysis of the *Arabidopsis* WAKL family RLKs was based on their protein sequences obtained from TAIR (The *Arabidopsis* Information Resource). A maximum likelihood phylogeny of WAKL proteins was generated with the program MEGA version 11.0.13, and their conserved domains were annotated using the NCBI Conserved Domain Database (https://www.ncbi.nlm.nih.gov/cdd/, accessed on 24 November 2025). The identification of WAKL10 protein domains was performed using the online programs SMART (Simple Modular Architecture Research Tool, https://smart.embl.de, accessed on 5 December 2025) and TMHMM-2.0 (https://services.healthtech.dtu.dk/services/TMHMM-2.0/, accessed on 5 December 2025).

### 2.4. Gene Expression Analysis

Following the manufacturer’s protocol, reverse transcription was carried out using 1 µg of total RNA and the PrimeScript RT Reagent Kit (Takara, Kusatsu, Shiga, Japan). RT-qPCR was then performed on a CFX96 Real-Time PCR Detection System (Bio-Rad, Hercules, CA, USA) with SYBR Green Real-time PCR Master mix (Toyobo, Osaka, Japan). The thermal cycling program was as follows: initial denaturation at 95 °C for 1 min; 39 cycles of 95 °C for 15 s, 60 °C for 15 s, and 72 °C for 45 s; followed by a final step at 65 °C for 5 s. A melting curve analysis was conducted after each run to verify amplicon specificity. Transcripts of *EF1α* (for *Arabidopsis*) and *ACT* (for tomato) served as internal reference genes. Relative transcript abundances were calculated using the 2−ΔΔCt method [31] and expressed as either fold induction relative to basal levels before treatment or as percentages. For each time point and genotype, three biological replicates were carried out, with each replicate consisting of either ten pooled *Arabidopsis* seedlings or three pooled tomato seedlings from independent GC vials. All primers used for qPCR are provided in Supplementary Table S1.

### 2.5. Ethylene Measurement

Using the same hydroponic culture system in GC vials described above, 14-day-old *Arabidopsis* or tomato seedlings were exposed to 10 µM Est, 100 nM flg22, 100 nM flgII-28, or their corresponding solvent controls. At designated time points following treatment, ethylene production was quantified by means of gas chromatography according to a previously described procedure [32]. Briefly, 1.5 mL of headspace gas was withdrawn from each sealed GC vial using a gas-tight syringe and injected into a gas chromatograph (PANNA A60, Beijing, China) with detector/injector/column oven temperatures set to 250 °C/250 °C/95 °C, respectively, and air/hydrogen/nitrogen flow rates at 400/40/25 mL/min. Ethylene concentration was calculated from standard curves and normalized to the fresh weight (FW) of seedlings. Three biological replicates (one GC vial containing 10 *Arabidopsis* or 3 tomato seedlings per replicate) were analyzed per time point and treatment.

### 2.6. Co-Immunoprecipitation and Immunoblotting Assays

*Arabidopsis* protoplasts were isolated and transformed with indicated plasmids as described previously [32]. After overnight incubation, protoplast transformants were treated with 100 nM flg22 for 10 min and then collected for co-IP assays. Co-IP experiments in protoplasts were independently repeated at least three times. Total protein extraction from protoplast transformants or transgenic seedlings and protein immunoprecipitation using anti-FLAG antibody-conjugated agarose, anti-BAK1 antibody plus protein A/G agarose, or anti-Myc antibody-conjugated agarose were all carried out as previously described [32]. Subsequent immunoblotting analyses using anti-HA, anti-FLAG, anti-Myc, anti-FLS2 or anti-BAK1 antibody were performed as described previously [32]. For in vivo co-IP assays in transgenic or mutant *Arabidopsis* plants, samples were prepared from three independent pools, each pool containing 10 *Arabidopsis* seedlings.

To assess MAPK activation triggered by flg22 or flgII-28, total protein was extracted from 14-day-old seedlings that had been exposed to 100 nM flg22 or 300 nM flgII-28 over a range of time points. Protein samples were then analyzed via immunoblotting using an anti-phospho-ERK1/2 (anti-pERK) antibody, following a previously reported protocol [32]. For each treatment condition and time point, samples were prepared as pooled mixtures consisting of either 10 *Arabidopsis* seedlings or 3 tomato seedlings.

### 2.7. ROS Burst Assay

ROS burst was measured via a luminol-based assay [33] with modifications. Leaf discs (5 mm diameter) were prepared from the third and fourth healthy true leaves of 4-week-old soil-grown *Arabidopsis* plants using a cork borer. For each genotype and treatment, ≥30 discs were prepared and placed individually into wells of a 96-well white microplate (Costar, Tewksbury, MA, USA) containing 100 μL deionized water (for wild-type/mutant) or 10 μM Est or DMSO (for Est-inducible lines). The plate was wrapped with plastic wrap and aluminum foil and incubated on an orbital shaker (low speed, room temperature, dark) for 24 h to attenuate wound responses and induce transgene expression. After incubation, the solution was replaced with 100 μL of freshly prepared elicitation mixture (100 nM flg22, 100 μM luminol, 10 μg/mL HRP; Sigma, St. Louis, MO, USA). Luminescence was recorded every 2 min for 60 min using a GloMax Navigator Microplate Luminometer (Promega, Madison, WI, USA); the readings are expressed as relative light units (RLU). From the ≥30 discs measured, 20 discs with consistent readings were selected as biological replicates. The mean and standard deviation were calculated, and the results are presented as both average kinetic curves and total RLU sums.

### 2.8. Pathogen Resistance Assay

Leaves of 4-week-old transgenic *Arabidopsis* or tomato plants were first infiltrated with either 10 μM Est or the solvent control (0.1% DMSO). After 24 h, the same leaves were inoculated with a bacterial suspension of *Pseudomonas syringae* pv. *tomato* (*Pst*) DC3000 (adjusted to OD_600_ = 0.0005 in 10 mM MgCl_2_) using a needleless syringe. Bacterial proliferation within the leaves was assessed at three days post-inoculation (dpi). For each genotype and treatment, three leaf discs (0.2 cm^2^ each) were collected from three independent plants (serving as biological replicates), pooled together, then serially diluted and plated on LB medium supplemented with 50 μg/mL rifampicin. Three technical replicates were plated for each dilution. The entire experiment was carried out twice independently, yielding comparable outcomes.

### 2.9. Tomato Transformation

Transgenic tomato plants were generated according to a previous report [34], with adjustments detailed below. Specifically, tomato (*S. lycopersicum* cv. Micro-Tom) leaf explants (5 mm × 5 mm) from 10-day-old seedlings were pre-cultured on T1 medium (MS salts, 3% sucrose, 2 mg/L zeatin, 0.1 mg/L IAA) for 1 d, incubated in an *Agrobacterium* suspension (OD_600_ = 0.3 in T1 medium supplemented with 100 μM acetosyringone) for 10 min, and then co-cultivated on the same T1 medium in darkness for 2 d. Explants were subsequently transferred to a selection medium (T1 medium containing 10 mg/L hygromycin and 200 mg/L timentin) for shoot regeneration under selective conditions. Shoots growing to 2–3 cm in height were excised and rooted on ½ MS medium supplemented with 3% sucrose, 2 mg/L IBA, and 100 mg/L timentin. Putative transgenic tomato plants were validated for HA-tagged protein expression by immunoblotting using anti-HA antibody. Two independent T1 lines were selected to propagate to the T2 generation; all assays were performed using T2 plants from at least two independent lines.

### 2.10. Statistical Analysis

Statistical analyses were conducted using GraphPad Prism version 9.5.1 or IBM SPSS Statistics version 26. All data are shown as mean ± standard deviation (SD). For experiments involving multiple biological replicates (*n* ≥ 3), the exact number of independent biological replicates (*n*) is provided in each figure legend. To determine significant differences, appropriate statistical tests were applied as indicated in the figure legends. These included two-way ANOVA followed by Tukey’s post hoc test for multiple comparisons, with significance denoted by lowercase letters (*p* < 0.05), and Student’s *t*-test for pairwise comparisons, with significance indicated by asterisks (* *p* < 0.05, ** *p* < 0.01, *** *p* < 0.001).

## 3. Results

### 3.1. WAKL10 Is the Most Strongly Induced WAKL Member in Response to flg22 Treatment

**Figure 1:**
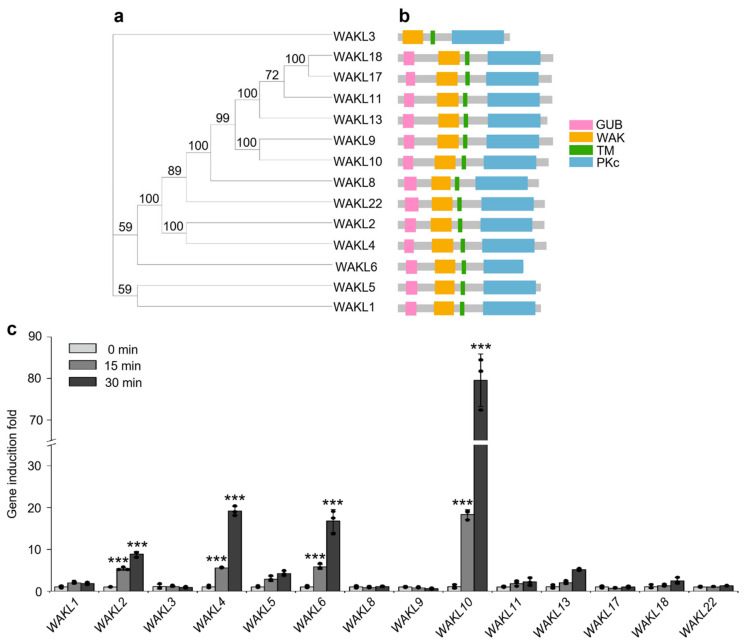
*WAKL10* transcript level is the highest in Arabidopsis upon flg22 treatment. (**a**) Phylogenetic analysis of the 14 *Arabidopsis WAKL* family RLKs that contain a canonical N-terminal WAK domain. A maximum likelihood (ML) tree was constructed using the full-length protein sequences of these 14 WAKL. Branch lengths are proportional to evolutionary distances. Bootstrap support values calculated using 5000 replicates are shown at nodes. (**b**) The conserved domain architectures of WAKL proteins. The extracellular galacturonan-binding (GUB) and WAK (yellow) domains, and the transmembrane (green) and intracellular kinase (blue) domains are indicated in different colors. (**c**) In *Arabidopsis*, flg22 treatment transcriptionally activates multiple *WAKL* genes. 14-day-old Col-0 wild-type seedlings grown in liquid half-strength MS medium were exposed to 100 nM flg22. At the indicated time points after flg22 addition, transcript levels of selected *WAKL* genes were measured via RT-qPCR and expressed as fold changes relative to their respective pre-treatment basal levels. Data are means ± SD (*n* = 3). Student’s *t*-test was used to compare each time point with its corresponding baseline; asterisks above the bars indicate statistical significance (*** *p* < 0.001). Black dots denote individual data points.

The *Arabidopsis* genome encodes 22 members of the Wall-Associated Kinase-Like (WAKL) family, among which 14 members contain the characteristic N-terminal WAK domain (Figure 1a,b and Figure S1). All these 14 WAKL members, except the putative WAKL3 protein, shared multiple conserved domains, including the extracellular galacturonan-binding (GUB) domain and the WAK domain, and the intracellular protein kinase (PKc) domain (Figure 1b). To investigate the potential roles of these 14 *WAKL* genes in flg22-triggered immunity, we performed RT-qPCR analyses to assess the expression patterns of these *WAKL* genes in response to flg22 treatment. As shown in Figure 1c, flg22 treatment highly induced the transcriptional expression of *WAKL2*, *WAKL4*, *WAKL6*, and *WAKL10*. Notably, *WAKL10* emerged as the most robustly induced *WAKL* member, with its transcript level peaking at 30 min post-treatment and showing an approximately 75-fold increase at this induction peak in comparison to the before-treatment basal level. This rapid and strong induction pattern of *WAKL10* suggests a prominent role of *WAKL10* in flg22-triggered immunity.

### 3.2. WAKL10 Positively Regulates flg22-Triggered Immunity

**Figure 2:**
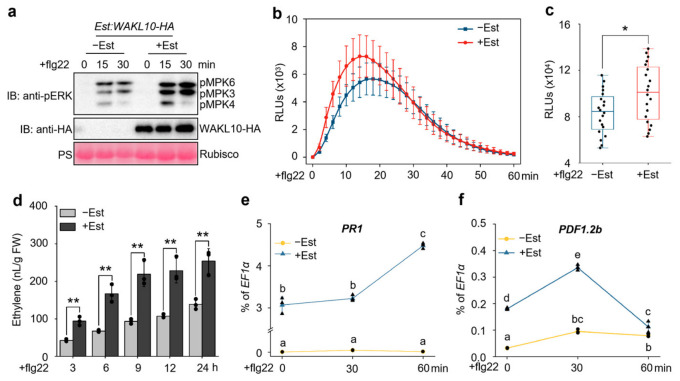
Estradiol-inducible expression of *WAKL10* in transgenic *Arabidopsis* plants potentiated flg22-triggered immune responses. (**a**,**d**–**f**) Pre-induction of WAKL10 by estradiol (Est) in *Est:WAKL10-HA* plants enhanced flg22-triggered defense responses, including MAPK activation (**a**), ethylene production (**d**), and upregulation of the defense marker genes *PR1* (**e**) and *PDF1.2b* (**f**). Fourteen-day-old *Est:WAKL10-HA* transgenic *Arabidopsis* seedlings grown in liquid half-strength MS medium were pre-treated with 10 μM Est or the solvent control (designated +/−Est) for 24 h, then challenged with 100 nM flg22 to induce PTI. At the indicated time points after flg22 application, activated MPK3, MPK4, and MPK6 together with Est-induced WAKL10-HA protein were detected via immunoblotting using anti-pERK and anti-HA antibodies, respectively; equal protein loading was verified via Ponceau S staining (PS) (**a**). Meanwhile, flg22-induced ethylene production was quantified by means of gas chromatography (**d**). Transcript levels of *PR1* and *PDF1.2b* were measured by RT-qPCR and expressed as percentages of the *EF1α* reference transcript (**e**,**f**). FW indicates fresh weight (**d**). All data are presented as means ± SD from three biological replicates (*n* = 3) (**d**–**f**). For panel (**d**), Student’s *t*-test was applied to determine significance between the compared datasets; asterisks denote ** *p* < 0.01. For panels (**e**,**f**), two-way ANOVA followed by Tukey’s HSD test was used to assess differences among all datasets; distinct lowercase letters indicate significant differences at *p* < 0.05. (**b**,**c**) The flg22-induced ROS burst in *Est:WAKL10-HA* plants was potentiated by Est-induced WAKL10. Leaf discs from 4-week-old soil-grown *Est:WAKL10-HA* plants were pretreated with 10 μM Est or solvent control (+/−Est) for 24 h and then treated with 100 nM flg22. Thereafter, ROS burst was detected every 2 min in 60 min using the luminol-HRP approach (**b**), and the cumulative ROS production in 60 min was quantified (**c**). Data are shown as mean ± SD (*n* = 20) (**b**,**c**). Asterisk indicates significant differences between the marked data (*, *p* < 0.05), as determined using Student’s *t*-test (**c**). RLUs, relative light units (**b**,**c**). Black dots or triangles represent individual data points (**c**–**f**).

To investigate the functional role of *WAKL10* in flg22-triggered immunity, we generated the estradiol (Est)-inducible *WAKL10*-overexpressing transgenic *Arabidopsis* plants (*Est:WAKL10-HA*), in which a HA-tagged WAKL10 protein could be induced by Est treatment (Figure 2a and Figure S2). We then examined whether *WAKL10* overexpression in *Est:WAKL10-HA* plants could enhance flg22-triggered immune responses. To do this, *Est:WAKL10-HA* seedlings were pretreated with Est to induce *WAKL10* expression and thereafter treated with flg22 to trigger PTI responses. As shown in Figure 2a, the Est-induced WAKL10 greatly augmented flg22-responsive activation of MPK3 and MPK6 in *Est:WAKL10-HA* plants. Pre-induction of WAKL10 also significantly potentiated flg22-induced ROS burst (Figure 2b,c) and ethylene biosynthesis (Figure 2d) in *Est:WAKL10-HA* plants. Moreover, the induction of PTI marker genes *PR1* and *PDF1.2b* in *Est:WAKL10-HA* plants after flg22 treatment was also largely enhanced by Est-induced WAKL10 (Figure 2e,f). Therefore, overexpression of *WAKL10* could strongly boost flg22-triggered immunity. Notably, before flg22 treatment, Est-mediated pre-induction of WAKL10 in *Est:WAKL10-HA* plants already led to high upregulation of *PR1* and *PDF1.2b* (Figure 2e,f), indicating that overexpressed *WAKL10* could induce *PR1* and *PDF1.2b* activation in the absence of flg22 elicitation. Together, these data indicate that overexpression of *WAKL10* can induce plant immune activation and promote flg22-triggered immunity.

**Figure 3:**
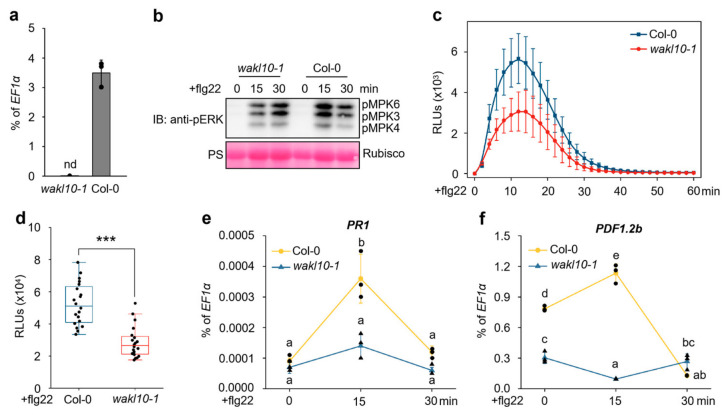
Flg22-triggered immune responses were attenuated in a *WAKL10* knockout mutant. (**a**) The *wakl10-1* mutant is a *WAKL10* knockout mutant. The transcript level of *WAKL10* in 2-week-old wild-type (Col-0) and *wakl10-1* mutant seedlings was determined via RT-qPCR and calculated as percentages of the *EF1α* reference transcript. Data are shown as mean ± SD (*n* = 3). nd, not detected. (**b**,**e**,**f**) In the *wakl10-1* mutant, flg22-induced MAPK activation (**b**) and upregulation of the defense marker genes *PR1* (**e**) and *PDF1.2b* (**f**) were reduced. 14-day-old wild-type (Col-0) and *wakl10-1* mutant seedlings grown in liquid half-strength MS medium were exposed to 100 nM flg22. At the indicated time points after flg22 treatment, activated MPK3, MPK4, and MPK6 were detected via immunoblotting using an anti-pERK antibody; equal protein loading was confirmed via Ponceau S staining (PS) (**b**). Separately, transcript levels of *PR1* and *PDF1.2b* were measured via RT-qPCR and expressed as percentages of the *EF1α* reference transcript (**e**,**f**). All data are presented as means ± SD from three biological replicates (*n* = 3) (**e**,**f**). For panels (**e**,**f**), two-way ANOVA followed by Tukey’s HSD test was applied to assess differences among all datasets; distinct lowercase letters indicate significant differences at *p* < 0.05. (**c**,**d**) The flg22-induced ROS burst was compromised in *wakl10-1* mutant. Leaf discs from 4-week-old soil-grown Col-0 and *wakl10-1* plants were treated with 100 nM flg22. ROS burst was detected every 2 min in 60 min by the luminol-HRP approach (**c**), and the cumulative ROS production in 60 min was quantified (**d**). Data are shown as mean ± SD (*n* = 20) (**c**,**d**). Asterisk indicates significant differences between the marked data (***, *p* < 0.001), as determined using Student’s *t*-test (**d**). RLUs, relative light units (**c**,**d**). Black dots or triangles represent individual data points (**a**,**d**–**f**).

To further validate the role of *WAKL10* in flg22-triggered immunity, we obtained a T-DNA insertional mutant of *WAKL10* (*wakl10-1*, SALK_132887), identified this mutant as a *WAKL10* knockout mutant (Figure 3a), and then characterized flg22-triggered immune responses in this mutant. As shown in Figure 3b–d, both flg22-triggered MPK3/MPK6 activation and ROS burst were attenuated in the *wakl10-1* mutant, compared with wild-type plants. The induction of PTI marker genes *PR1* and *PDF1.2b* by flg22 was also compromised in *wakl10-1*, particularly at 15 min after flg22 treatment (Figure 3e,f), while flg22-induced ethylene production was not impaired in *wakl10-1* (Figure S3). Collectively, our gain-of-function and loss-of-function genetic analyses identified *WAKL10* as a positive regulator that promotes flg22-triggered immunity.

### 3.3. WAKL10 Promotes FLS2-BAK1 Receptor Complex Assembly in Arabidopsis

**Figure 4:**
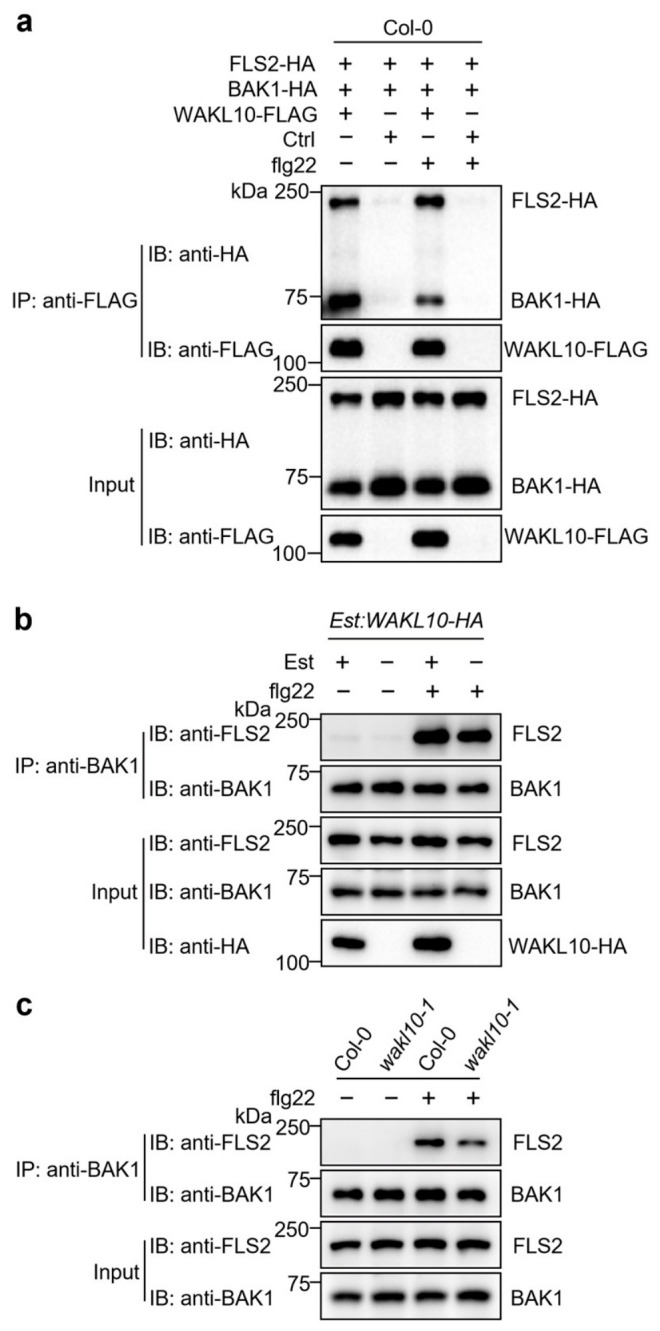
WAKL10 associates with FLS2 and BAK1 to promote the flg22-induced FLS2-BAK1 complex formation. (**a**) WAKL10 interacts with both FLS2 and BAK1. WAKL10-FLAG was co-expressed together with FLS2-HA and BAK1-HA in *Arabidopsis* (Col-0) protoplasts. The transformed protoplasts were then exposed to 100 nM flg22 or a ddH_2_O control (+/−flg22) for 10 min. After immunoprecipitation (IP) using anti-FLAG agarose beads, the recovered proteins were subjected to immunoblotting (IB) with anti-HA or anti-FLAG antibodies (top two panels). Input protein levels were visualized via immunoblotting with the indicated antibodies (bottom two panels). Ctrl denotes the vector control. (**b**,**c**) WAKL10 facilitates the flg22-induced association between FLS2 and BAK1. For panel (**c**), 14-day-old Col-0 and *wakl10-1* seedlings grown in liquid half-strength MS medium were exposed to 100 nM flg22 or a ddH_2_O control (+/−flg22) for 15 min. For panel (**b**), *Est:WAKL10-HA* transgenic seedlings maintained under the same conditions were first pre-treated with 10 μM Est or the solvent control (+/−Est) for 24 h, and then challenged with 100 nM flg22 or ddH_2_O control for an additional 15 min. Following immunoprecipitation (IP) from total seedling proteins using protein A/G agarose beads conjugated with anti-BAK1 antibody, the recovered proteins were subjected to immunoblotting (IB) with anti-BAK1 or anti-FLS2 antibodies (top two panels). Input protein levels were visualized via immunoblotting with the indicated antibodies (bottom panels).

Flg22 is perceived by the receptor FLS2 and co-receptor BAK1 [35]. Upon flg22 binding, FLS2 rapidly recruits BAK1 to form an active receptor complex, which subsequently initiates a series of intracellular PTI signaling events, such as activation of MAPK cascades, production of reactive oxygen species, and induction of ethylene biosynthesis [36]. To explore the mechanism of WAKL10-mediated potentiation of flg22-triggered immunity, we investigated whether WAKL10 physically associates with the FLS2-BAK1 receptor complex. Co-immunoprecipitation (co-IP) assays in *Arabidopsis* protoplasts showed that WAKL10 constitutively interacts with both FLS2 and BAK1, regardless of flg22 elicitation (Figure 4a), suggesting that WAKL10 forms pre-existing protein complexes with FLS2 and BAK1 independently of ligand perception. We then examined whether WAKL10 could modulate the formation of FLS2-BAK1 complex in response to flg22 elicitation using *Est:WAKL10-HA* transgenic plants and *wakl10-1* mutant. As shown in Figure 4b, based on co-IP assays, the abundance of flg22-induced FLS2-BAK1 complexes was slightly increased upon Est-mediated pre-induction of WAKL10 in *Est:WAKL10-HA* plants. By contrast, the induction of FLS2-BAK1 complexation by flg22 was compromised in *wakl10-1* mutant compared with wild-type plants (Figure 4c). Taken together, these data indicate that WAKL10 constitutively associates with FLS2 and BAK1, and promotes flg22-triggered immunity by facilitating the assembly of the FLS2-BAK1 complex.

### 3.4. The Function of WAKL10 in Promoting flg22-Triggered Immunity Is Independent of Its Intracellular Kinase Domain

**Figure 5:**
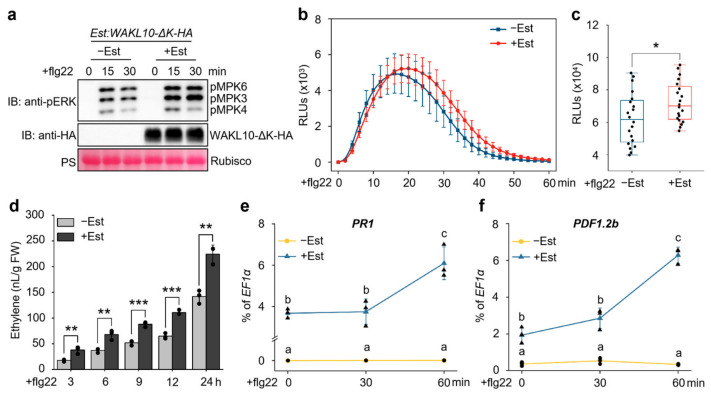
The intracellular kinase domain of WAKL10 is not required for its function in promoting flg22-triggered immunity. (**a**,**d**–**f**) Pre-induction of a kinase domain-deleted WAKL10 mutant (WAKL10-ΔK) by estradiol (Est) in *Est:WAKL10-ΔK-HA* transgenic plants enhanced flg22-triggered defense outputs, including MAPK activation (**a**), ethylene production (**d**), and upregulation of the defense marker genes *PR1* (**e**) and *PDF1.2b* (**f**). 14-day-old *Est:WAKL10-ΔK-HA* transgenic seedlings grown in liquid half-strength MS medium were pre-treated with 10 μM Est or the solvent control (designated +/−Est) for 24 h and then challenged with 100 nM flg22 to induce PTI. At the indicated time points after flg22 application, activated MPK3, MPK4, and MPK6 together with the Est-induced WAKL10-ΔK-HA protein were detected via immunoblotting using anti-pERK and anti-HA antibodies, respectively; equal protein loading was verified via Ponceau S staining (PS) (panel (**a**)). Concurrently, flg22-induced ethylene production was quantified by means of gas chromatography (panel (**d**)). Transcript levels of *PR1* and *PDF1.2b* were measured via RT-qPCR and expressed as percentages of the *EF1α* reference transcript (panels (**e**,**f**)). FW denotes fresh weight (panel (**d**)). All data are presented as means ± SD from three biological replicates (*n* = 3) (panels (**d**–**f**)). For panel (**d**), Student’s *t*-test was used to evaluate significance between the compared datasets; asterisks indicate ** *p* < 0.01 and *** *p* < 0.001 (**d**). For panels (**e**,**f**), two-way ANOVA followed by Tukey’s HSD test was applied to assess differences among all datasets; distinct lowercase letters indicate significant differences at *p* < 0.05. (**b**,**c**) The flg22-induced ROS burst in *Est:WAKL10-ΔK-HA* plants was potentiated by Est-induced WAKL10-ΔK. Leaf discs from 4-week-old soil-grown *Est:WAKL10-ΔK-HA* plants were pretreated with 10 μM Est or solvent control (+/−Est) for 24 h and then treated with 100 nM flg22. Thereafter, ROS burst was detected every 2 min in 60 min by the luminol-HRP approach (**b**), and the cumulative ROS production in 60 min was quantified (**c**). Data are shown as mean ± SD (*n* = 20) (**b**,**c**). Asterisk indicates significant differences between the marked data (*, *p* < 0.05), as determined using Student’s *t*-test (**c**). RLUs, relative light units (**b**,**c**). Black dots or triangles represent individual data points (**c**–**f**).

Receptor-like kinases (RLKs) typically depend on extracellular domains for signal perception and intracellular kinase domains for signal transduction [37]. WAKL10 possesses a canonical RLK structure, comprising an extracellular domain, transmembrane region, and intracellular kinase domain (Figure S4). To identify whether the kinase domain of WAKL10 is involved in regulating flg22-triggered immunity, we generated a kinase domain-deleted mutant of WAKL10 (WAKL10-ΔK) and analyzed its interaction with FLS2 and BAK1 in *Arabidopsis* protoplast via co-IP assays. As shown in Figure S5, similar to full-length WAKL10, the WAKL10-ΔK mutant also constitutively interacted with FLS2 and BAK1 independently of flg22 elicitation, indicating that the kinase domain of WAKL10 is not involved in its interaction with FLS2 and BAK1. To further evaluate the involvement of WAKL10 kinase domain in flg22-triggered immunity, the Est-inducible *WAKL10-ΔK*-overexpressing transgenic *Arabidopsis* plants (*Est:WAKL10-ΔK-HA*) were generated (Figure S6), then pretreated with Est to induce *WAKL10-ΔK* expression, and thereafter treated with flg22 to characterize PTI responses. As shown in Figure 5, Est-mediated pre-induction of WAKL10-ΔK significantly potentiated flg22-induced MPK3/MPK6 activation (Figure 5a) and ROS burst (Figure 5b,c) in *Est:WAKL10-ΔK-HA* plants. The induction of ethylene biosynthesis (Figure 5d) and the upregulation of PTI marker genes *PR1* (Figure 5e) and *PDF1.2b* (Figure 5f) in *Est:WAKL10-ΔK-HA* plants after flg22 treatment were also largely enhanced by Est-induced WAKL10-ΔK. These results indicate that WAKL10-ΔK retains the function of wild-type WAKL10 in promoting flg22-triggered immunity. Collectively, these data indicate that the intracellular kinase domain of WAKL10 is likely not required for its function in flg22-triggered immunity, and suggest that WAKL10 probably relies on its extracellular domain to promote flg22-triggered immunity by facilitating the assembly of the FLS2-BAK1 complex.

### 3.5. Overexpression of WAKL10 in Arabidopsis and Tomato Confers Enhanced Resistance to the Bacterial Pathogen Pst DC3000

**Figure 6:**
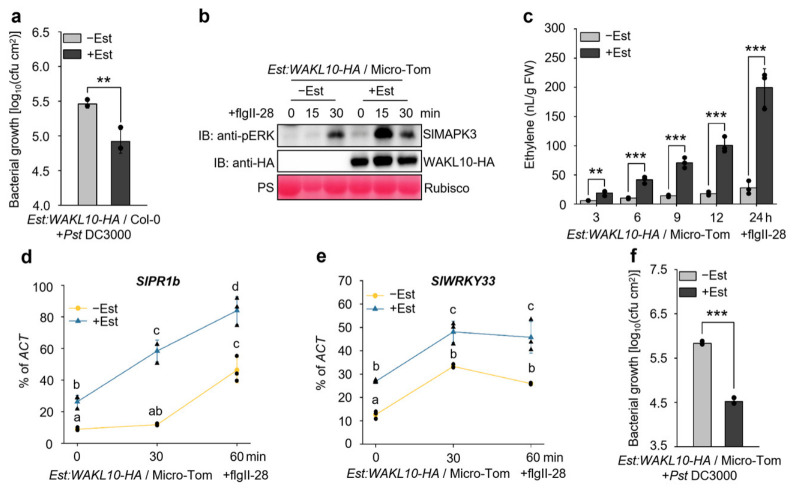
Estradiol-inducible expression of *WAKL10* in transgenic *Arabidopsis* and tomato plants enhanced disease resistance against the bacterial pathogen *Pst* DC3000. (**a**,**f**) In both *Arabidopsis* and tomato, pre-induction of WAKL10 expression in *Est:WAKL10-HA* transgenic plants enhanced resistance against *Pst* DC3000. Mature leaves of 4-week-old *Est:WAKL10-HA* plants (*Arabidopsis* or tomato) were first infiltrated with 10 μM Est or the solvent control (+/−Est). One day later, the same leaves were inoculated with *Pst* DC3000. Bacterial proliferation was measured three days post-inoculation. (**b**–**e**) In tomato, Est-driven WAKL10 pre-expression also potentiated flgII-28-triggered defenses, including MAPK activation (**b**), ethylene production (**c**), and upregulation of the defense genes *SlPR1b* (**d**) and *SlWRKY33* (**e**). Two-week-old *Est:WAKL10-HA* tomato seedlings were pre-treated with 10 μM Est or solvent control (+/−Est) in liquid medium for 24 h, then challenged with 300 nM flgII-28. At the indicated time points after flgII-28 application, activated MAPKs and the induced WAKL10-HA protein were detected via immunoblotting using anti-pERK and anti-HA antibodies, respectively; equal protein loading was verified via Ponceau S staining (PS) (panel (**b**)). Concurrently, flgII-28-induced ethylene production was quantified by means of gas chromatography (panel (**c**)). Transcript levels of *SlPR1b* and *SlWRKY33* were measured via RT-qPCR and expressed as percentages of the *ACT* reference transcript (panels (**d**,**e**)). FW denotes fresh weight (panel (**c**)). All data are presented as means ± SD from three biological replicates (*n* = 3) (panels (**a**,**c**–**f**)). Student’s *t*-test was used to assess significance between the indicated datasets (panels (**a**,**c**,**f**)); asterisks indicate ** *p* < 0.01 and *** *p* < 0.001. Two-way ANOVA followed by Tukey’s HSD test was applied to evaluate differences among all datasets (panels (**d**,**e**)); distinct lowercase letters denote significant differences at *p* < 0.05. Black dots or triangles represent individual data points (panels (**a**,**c**–**f**)).

Given the function of *WAKL10* in promoting flg22-triggered immunity, we further evaluated the potential application of *WAKL10* in engineering plant disease resistance. To this end, we firstly examined whether pre-induction of WAKL10 in *Est:WAKL10-HA* transgenic *Arabidopsis* plants could enhance disease resistance against the bacterial pathogen *Pst* DC3000. As shown in Figure 6a, the resistance of *Est:WAKL10-HA Arabidopsis* plants against *Pst* DC3000 was significantly enhanced by Est-induced WAKL10. Consistently, a previous study showed that mutation of *WAKL10* led to increased susceptibility to *Pst* DC3000 [38], further supporting the contribution of *WAKL10* to *Arabidopsis* bacterial resistance. Cross-species transfer of immune receptors represents a promising strategy for engineering disease resistance in crops [39]. The efficacy of *WAKL10* in improving *Arabidopsis* bacterial resistance prompted us to further explore its applications in engineering disease-resistant crops.

To investigate whether *WAKL10* overexpression could enhance immune responses and disease resistance in tomato crop (*S. lycopersicum*), we transformed the *Est:WAKL10-HA* construct into the tomato cultivar Micro-Tom to generate Est-inducible *WAKL10*-overexpressing transgenic tomato plants (Figure S7). As shown in Figure 6b,c, Est-mediated pre-induction of WAKL10 in *Est:WAKL10-HA* transgenic tomato plants strongly potentiated MAPK activation and ethylene induction in response to the treatment of flgII-28, another flagellin epitope that can be detected by tomato [40]. The flgII-28-induced upregulation of defense marker genes *SlPR1b* and *SlWRKY33* was also largely elevated by Est-induced WAKL10 in *Est:WAKL10-HA* tomato plants (Figure 6d,e). Consistent with WAKL10-mediated potentiation of flgII-28-triggered immune responses, the resistance of *Est:WAKL10-HA* tomato plants against the *Pst* DC3000 was also significantly enhanced upon pre-induction of WAKL10 by Est treatment (Figure 6f). Thus, overexpression of *WAKL10* in tomato can also boost flgII-28-triggered immunity and suggesting its potential application in engineering crop plants for improved disease resistance. However, we recognize that further evaluation under field conditions and in additional tomato cultivars is required to assess its practical utility.

## 4. Discussion

The WAK/WAKL family of RLKs are generally considered to bind plant cell wall-derived pectin or pectin fragments to regulate plant development and defense responses [41,42,43]. In *Arabidopsis*, several WAKs, including WAK1, WAK2 and WAK4, interact with native pectin in plant cell walls to regulate cell expansion [44,45], while WAKL22, also called RFO1 (RESISTANCE TO FUSARIUM OXYSPORUM 1), binds de-methylated pectin and acts as a sensor of the pectin methylation status in plant cell walls to regulate defense against the fungal vascular pathogen *Fusarium oxysporum* [46,47]. In addition, *Arabidopsis* WAK1 also perceives pectin fragments of oligogalacturonide (OG) released from plant cell walls during insect or pathogen attack to trigger immune responses [29]. In this study, among the *WAKL* family genes, *WAKL10* was identified as the most strongly induced *WAKL* member in *Arabidopsis* upon treatment with the bacterial PAMP flg22 (Figure 1c). Subsequent genetic and biochemical analyses indicate that WAKL10 associates with both the flg22 receptor FLS2 and the co-receptor BAK1 to promote flg22-triggered immunity (Figure 2, Figure 3 and Figure 4). Although a complementation line in the *wakl10-1* background is not yet available, the convergent evidence from both the loss-of-function mutant and overexpression lines consistently supports a positive role for *WAKL10* in flg22-triggered immunity. Interestingly, the intracellular kinase domain of WAKL10 is not required for its function in promoting flg22-triggered immunity, suggesting that WAKL10 probably depends on its extracellular domain to promote flg22-triggered immunity by facilitating the assembly of the FLS2-BAK1 complex. Thus, this study reveals a new mechanism of WAKLs in regulating plant immunity, i.e., via promoting the assembly of LRR-RLK-type PRR complex. This also represents a new function of WAKLs in plant immunity, which is distinguished from their proposed roles in sensing cell wall components such as pectin fragments.

Expression of *WAKL10* or *WAKL10-ΔK* alone increased *PR1* and *PDF1.2b* transcript levels (Figure 2e,f; Figure 5e,f), indicating that overexpression primes or partially activates defense responses. This basal activation may facilitate a more rapid potentiation of flg22-triggered immunity, as flg22 treatment further elevated these transcripts beyond the basal level. Nevertheless, the solvent control (−Est) under identical flg22 treatment allows us to distinguish this basal priming from the specific potentiation of flg22 signaling. Loss of *WAKL10* does not uniformly impair flg22 responses: ethylene production is unaffected despite reduced MAPK activation and ROS burst (Figure S3; Figure 3b–d). This selective defect may reflect pathway-specific regulation by *WAKL10*, which could preferentially promote certain signaling branches. Alternatively, functional redundancy among flg22-induced WAKL family members may compensate for *WAKL10* loss in the ethylene pathway. Future studies using higher-order WAKL mutants are needed to test these possibilities.

Moreover, overexpression of the kinase-deletion mutant (*WAKL10-ΔK*) resulted in a weaker flg22-triggered immune response compared to full-length WAKL10, suggesting that the kinase domain might play a subtle regulatory role not captured by WAKL10-ΔK, and that the increased FLS2-BAK1 complex abundance upon *WAKL10* overexpression could result from indirect mechanisms, such as stabilization of the receptor complex at the plasma membrane. Given the functional versatility of the WAK/WAKL family, with some members sensing pectin [44,45] and others promoting PRR complex assembly [48,49] *WAKL10* may have evolved a specialized function in flagellin-triggered immunity. Future comparative studies across WAKL family members are required to dissect these possibilities.

In addition to *Arabidopsis* WAKL10, a tomato member of the WAK family, SlWAK1, has also been linked to flagellin-triggered immunity. Its transcript abundance rises strongly following exposure to the flagellin-derived PAMPs flg22 and flgII-28 [50], which are recognized respectively by the tomato PRRs SlFLS2 and SlFLS3 [35,51]. Similar to *Arabidopsis* WAKL10, SlWAK1 associates with FLS2 and FLS3 regardless of flg22 or flgII-28 presence, and it is essential for late PTI responses induced by these two elicitors in tomato [48]. Furthermore, the cotton GhWAK7A has been demonstrated to interact with the chitin receptors GhLYK5 and GhCERK1, facilitating their chitin-induced dimerization and thereby playing a critical role in chitin-triggered immunity as well as resistance to fungal wilt disease in cotton [49]. Thus, these previous reports together with this study suggest that WAK/WAKLs may broadly interact with bona fide PRR receptors and promote PTI responses and disease resistance, possibly by facilitating the assembly of PRR receptor complexes. Future studies are required to systematically identify and elucidate the roles of WAK/WAKL family RLKs in promoting PRR complex formation and PTI-mediated pathogen resistance.

In addition to WAKs/WAKLs, several other RLKs from *Catharanthus roseus* receptor-like kinase 1-like (CrRLK1L) or LRR-RLK family have also been shown to interact with FLS2 and/or BAK1 to promote flg22-induced immunity and disease resistance. Similar to WAKL10, the CrRLK1L family RLK FERONIA (FER), containing extracellular malectin-like domains, also interacts with FLS2 and BAK1 to facilitate flg22 perception [52]. In addition, IOS1 (IMPAIRED OOMYCETE SUSCEPTIBILITY1), an RLK containing both extracellular malectin-like and LRR domains but not belonging to the CrRLK1L family, also associates with FLS2 and BAK1 to positively regulate flg22 signaling [53]. Therefore, multiple RLKs associate with FLS2 and/or BAK1 to facilitate flg22 perception and signaling. Future multidisciplinary studies are needed to decipher how these RLKs are dynamically organized and coordinated to modulate flg22-induced FLS2-BAK1 receptor complex assembly and regulate flg22-triggered immunity.

## Data Availability

The data that support the findings of this study are available from the corresponding author upon reasonable request.

## Supplementary Materials

The following supporting information can be downloaded at https://www.mdpi.com/article/10.3390/genes17050561/s1. Figure S1: Phylogenetic analysis of the WAKL family RLKs in *Arabidopsis*; Figure S2: Est-induced expression of WAKL10 in *Est:WAKL10-HA* transgenic *Arabidopsis* lines; Figure S3: The flg22-induced ethylene production level in the *wakl10-1* mutant is comparable to that in wild-type Col-0 plants; Figure S4: A schematic diagram showing the domain architecture of WAKL10 and its kinase domain-deleted mutant (WAKL10-ΔK); Figure S5: The intracellular kinase domain of WAKL10 is not required for its association with FLS2 and BAK1; Figure S6: Est-induced expression of WAKL10-ΔK in *Est:WAKL10-ΔK-HA* transgenic *Arabidopsis* lines; Figure S7: Est-induced expression of WAKL10 in *Est:WAKL10-HA* transgenic tomato lines. Table S1: Primers used for RT-qPCR analyses in this study; Table S2: Primers used for the generation of recombinant constructs in this study.
