# Supplementary material for: The WAKL10 Gene Promotes Flg22-Triggered Immunity by Interacting with FLS2 and BAK1 in Arabidopsis
Source: Genes (Basel). 2026 May 9;17(5):561. doi: 10.3390/genes17050561 (PMC13205364; doi:10.3390/genes17050561)
Supplement: Supplementary file 1 [file genes-17-00561-s001.zip › genes-4271781-supplementary.pdf]

Supplementary Information (SI)

The *WAKL10* Gene Promotes Flg22-Triggered Immunity by Interacting with FLS2 and BAK1 in *Arabidopsis*

Authors: Lu Zhang[1,2], Jiale Gao[2], Lingya Yao[2]†, Yunxia He[2]†

Affiliations:

[1] School of Environmental and Chemical Engineering, Shanghai University, Shanghai 200444, China.

[2] Shanghai Key Laboratory of Plant Molecular Sciences, College of Life Sciences, Shanghai Normal University, Shanghai 200234, China.

† Correspondence: yaolingya@shnu.edu.cn (L.Y.); yxhe@shnu.edu.cn (Y.H.)

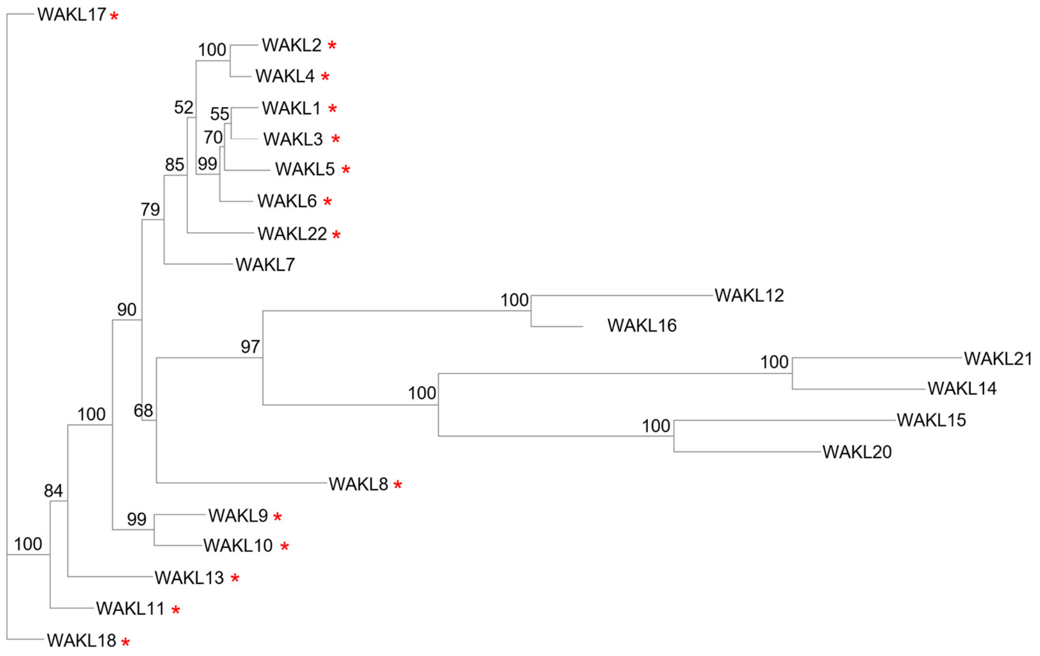

Figure S1. Phylogenetic analysis of the WAKL family RLKs in *Arabidopsis*
A maximum likelihood (ML) tree was constructed using the full-length protein sequences of 21 WAKL members (WAKL19 was excluded due to the lack of a confirmed genomic locus). Branch lengths are proportional to evolutionary distances. Bootstrap support values calculated using 5,000 replicates are shown at nodes. Red asterisks indicate members containing a canonical N-terminal WAK domain.

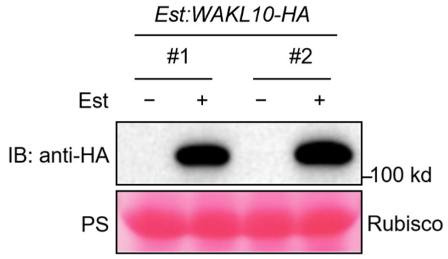

Figure S2. Est-induced expression of WAKL10 in *Est:WAKL10-HA* transgenic *Arabidopsis* lines

Two-week-old *Est:WAKL10-HA* transgenic *Arabidopsis* seedlings cultured in liquid ½ MS media were treated with 10 μM Est or solvent control (+/−Est) for 24 h. The induced WAKL10-HA protein was detected by immunoblotting using anti-HA antibody (IB: anti-HA). Total protein loading was assessed by Ponceau S staining (PS).

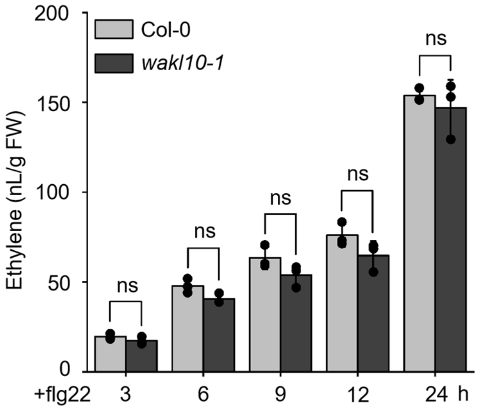

Figure S3. The flg22-induced ethylene production level in *wakl10-1* mutant is comparable to that in wild-type Col-0 plants

Two-week-old wild-type (Col-0) and *wakl10-1* mutant seedlings cultured in liquid ½ MS media were treated with 100 nM flg22. At indicated time points after flg22 treatment, the ethylene induction was measured by gas chromatography. Data are shown as mean ± SD (n = 3). ns, no significant difference between the marked data, as determined by Student's t-test. Black dots represent individual data points.

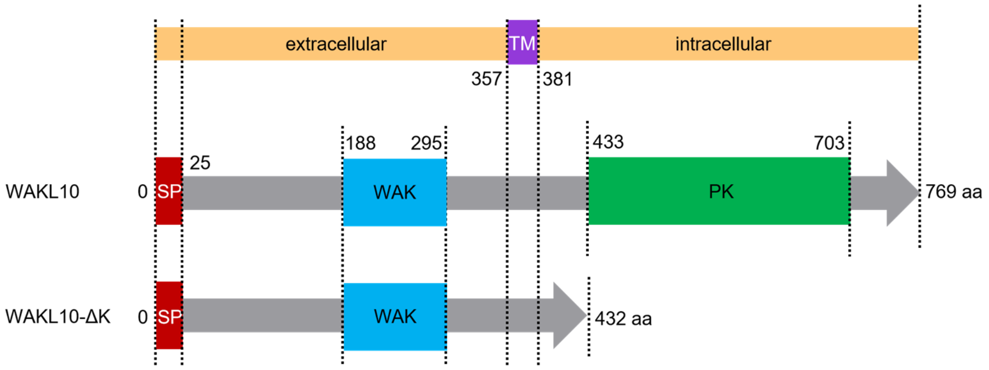

Figure S4. A schematic diagram showing the domain architecture of WAKL10 and its kinase domain-deleted mutant (WAKL10-ΔK)

The WAKL10 protein domains were identified using the online programs SMART (https://smart.embl.de) and TMHMM-2.0 (https://services.healthtech.dtu.dk/services/TMHMM-2.0/). The positions of different domains in WAKL10 protein are labeled. SP, signal peptide; PK, protein kinase; TM, transmembrane domain.

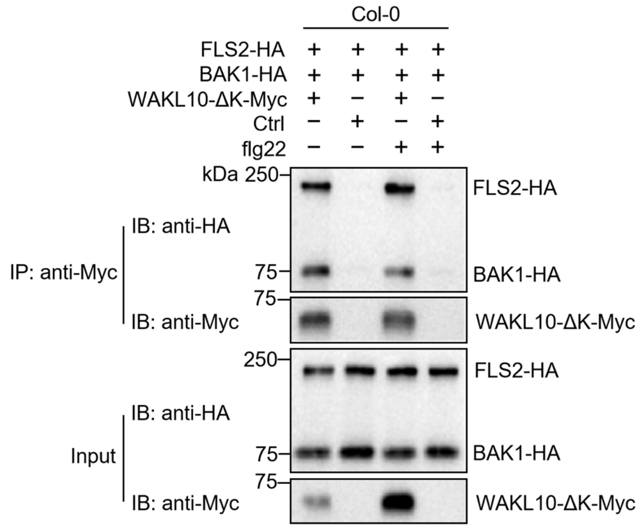

Figure S5. The intracellular kinase domain of WAKL10 is not required for its association with FLS2 and BAK1

The association of a kinase domain-deleted WAKL10 mutant (WAKL10-ΔK) with FLS2 or BAK1 was analyzed via co-IP assays in *Arabidopsis* protoplasts. To do this, *Arabidopsis* Col-0 protoplasts were transfected with the constructs expressing WAKL10-ΔK-Myc together with FLS2-HA or BAK1-HA. Protoplast transformants were treated with 100 nM flg22 or ddH_2O control (+/−flg22) for 10 min. The proteins immunoprecipitated from protoplast total proteins using anti-Myc agarose beads (IP: anti-Myc) were analyzed by immunoblotting with anti-HA (IB: anti-HA) or anti-Myc antibody (IB: anti-Myc) (top two panels). The protein inputs were shown by immunoblotting with the indicated antibodies (bottom two panels). Ctrl: vector control.

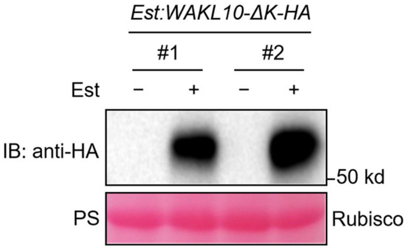

Figure S6. Est-induced expression of WAKL10-ΔK in *Est:WAKL10-ΔK-HA* transgenic *Arabidopsis* lines

Two-week-old *Est:WAKL10-ΔK-HA* transgenic *Arabidopsis* seedlings cultured in liquid ½ MS media were treated with 10 µM Est or solvent control (+/−Est) for 24 h. The induced WAKL10-ΔK-HA protein was detected by immunoblotting using anti-HA antibody (IB: anti-HA). Total protein loading was assessed by Ponceau S staining (PS).

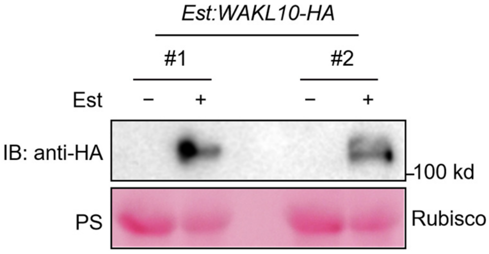

Figure S7. Est-induced expression of WAKL10 in *Est:WAKL10-HA* transgenic tomato lines

Two-week-old *Est:WAKL10-HA* transgenic tomato seedlings cultured in liquid ½ MS media were treated with 10 μM Est or solvent control (+/−Est) for 24 h. The induced WAKL10-HA protein was detected by immunoblotting using anti-HA antibody (IB: anti-HA). Total protein loading was assessed by Ponceau S staining (PS).

Table S1. Primers used for RT-qPCR analyses in this study

Primer Name	Sequence
WAKL1-RT-F	CAGGCCTAACCCCAAGATAAC
WAKL1-RT-R	ATAGCCAGAATAGCCCAACAAA
WAKL2-RT-F	GATCAAAGCTGTGTGAATCTGC
WAKL2-RT-R	GAAAAGCAACAGTGCTGAACCT
WAKL3-RT-F	AGGCAATGAAAGAGAACAGAGC
WAKL3-RT-R	ATCTTCTGGCGAGTTTTGCTAC
WAKL4-RT-F	GCAAAGACCAAAGTTGTGTGAA
WAKL4-RT-R	GTACAACCCGAAAATTCCAAAG
WAKL5-RT-F	TCTGTGAAGAAGGCACTTGTGT
WAKL5-RT-R	AAGCGTCCCAACAACTAAGAAC
WAKL6-RT-F	AGATAACGAAACCGGAAAAAGC
WAKL6-RT-R	TGATCCTCGTTCGCTTCTTAAT
WAKL8-RT-F	GATGAATGCAGAGATCCACATC
WAKL8-RT-R	ACCAAAGATCAGGAGCAACAAT
WAKL9-RT-F	AAGAGGGCATGACTTACTGTGG
WAKL9-RT-R	GATCAATGAGCCAAAACTAGCA
WAKL10-RT-F	GGCGGTTATACGTGTGAGTACA
WAKL10-RT-R	ACCAATGAATACCAATGTGCTG
WAKL11-RT-F	ACGCCAAAATTGTAGAGAAAGC
WAKL11-RT-R	AGGACTAAGATGCCAAAAGCAG
WAKL13-RT-F	TAATACATCGGGAGGTCATCGT
WAKL13-RT-R	AAGAAGTTTTCTCCACCACCAT
WAKL17-RT-F	TGGGCGATAAGACTAAAGCAAT
WAKL17-RT-R	CTTCTTCTTCCTCTTTGCCATC
WAKL18-RT-F	TGAATTTTGAAGGAGGCTATCG
WAKL18-RT-R	CCACCATAATCCACCAACTAGG
WAKL22-RT-F	ACTTGTGTGAATGTTCCTGGAT
WAKL22-RT-R	ACGAACTTGATCAATCCCCATA
EF1α-RT-F	TGAGCACGCTCTTCTTGCTTTCA
EF1α-RT-R	GGTGGTGGCATCCATCTTGTTACA
PR1-RT-F	ATGAATTTTACTGGCTATTCTCGATT
PR1-RT-R	CTGCATGGGACCTACGCCTACCGCTCC
PDF1.2b-RT-F	AGTTTGCTTCCATCATCACCTT
PDF1.2b-RT-R	AAGATCCATGTTTTGCTCCTTC
SlPR1b-RT-F	ATTCATTCTGGTGCTGGGGA
SlPR1b-RT-R	GTTGCGCCAGACTACTTGAG
SlWRKY33-RT-F	GAGATGGAAGGGTGACAATGAA
SlWRKY33-RT-R	GGTTCGGATTTCCTTTGACAA
ACT-RT-F	TGAGCTTCGAGTTGCTCCTGA
ACT-RT-R	AGCACAGCCTGGATAGCAACA

Table S2. Primers used for generation of recombinant constructs in this study

Primer Name	Sequence
WAKL10(CDS)-F-SalI-BglII	ACGCGTCGACAGATCTATGAGCTCTAATTGTAGTTG TTCTCTT
WAKL10(CDS)-R-XbaI-StuI	GCTCTAGAAGGCCTCCGAGGAAACAATGGTTCA
WAKL10(CDS-ΔK)-R-XbaI-StuI	GCTCTAGAAGGCCTGTTTTCGGTGGCTTTCTCCA
FLS2(CDS)-F-SalI-BamHI	ACGCGTCGACGGATCCATGAAGTTACTCTCAAAGA CCTTTTTGAT
FLS2(CDS)-R-SpeI-SmaI	GGACTAGTCCCGGGAACTTCTCGATCCTCGTTACGA TC
BAK1(CDS)-F-XhoI-BamHI	CCGCTCGAGGGATCCTGGAACGAAGATTAATGATC CCTT
BAK1(CDS)-R-XbaI-StuI	GCTCTAGAAGGCCTTCTTGGACCCGAGGGGTATT
